# RHD Genotyping by Molecular Analysis of Hybrid *Rhesus box* in RhD-Negative Blood Donors from Iran

**DOI:** 10.1007/s12288-018-0992-3

**Published:** 2018-08-01

**Authors:** Behzad Nazel Khosroshahi, Arezoo Oodi, Saba Namjou, Tahereh Gholamali, Naser Amirizadeh

**Affiliations:** Blood Transfusion Research Center, High Institute for Research and Education in Transfusion Medicine, Hemmat Highway, Next to the Milad Tower, Tehran, Iran

**Keywords:** *RHD* genotype, Hybrid *Rhesus box*, *RHD* gene alleles, RhD-negative donors, Iran

## Abstract

D antigen is the most important and immunogenic antigen of the Rh blood group. The RhD-negative phenotype has different genetic backgrounds with variable distribution in different populations. Hybrid *Rhesus box*, resulting from *RHD* gene deletion, is used in genotyping studies of the Rh blood group as a marker to identify the *RHD* gene deletion. This study for the first time identified genetic mechanisms for the occurrence of RhD-negative phenotype among the Iranian population. 200 RhD-negative blood donors were randomly selected from Tehran Blood Transfusion Center. The phenotype of D, C, Ε, e and c antigens was serologically identified, and DNA was extracted from buffy coat. The molecular analysis of hybrid *Rhesus box* was performed by PCR-SSP and PCR-RFLP. Moreover, the presence of different exons of *RHD* gene was investigated by real-time PCR on extracted DNA. Hybrid *Rhesus box* was detected in all samples, and PCR-RFLP confirmed that 198 (99%) were *homozygous* for an *RHD* gene deletion and 2 were *heterozygous* for hybrid *Rhesus box* in which one (0.5%) had a weak D type 11 and the other one (0.5%) had a *RHD*-*CE (2*-*9)*-*D*_*2*_ hybrid allele. Similar to Caucasians, the frequency of *RHD* gene deletion was high among the Iranian population studied in this investigation, so hybrid *Rhesus box* can be used as an efficient marker to detect *RHD* gene deletion in our population.

## Introduction

The antigen D is the most important and immunogenic antigen of the Rh blood group [1, 2]. The proper identification of RhD antigen has a high clinical significance, and can play an important role in preventing the occurrence of hemolytic anemia in patients and newborns [2]. Serology is the standard method for detection of RhD antigen, but has limitations. Genotyping by molecular techniques is a complementary tool to overcome these limitations. Some of the clinical applications of these methods are determination of *RHD* zygosity in RhD-positive fathers, assessment of RhD status in the fetus and multi-transfused patients, resolving the discrepancies in RhD typing in blood donors and patients with auto or alloantibodies [3, 4]. Knowing the prevalence of alleles responsible for the phenotype in a special population, and the establishment of appropriate molecular methods for detection of alleles are required for genotyping and its application in the clinic [5]. Whereas in Caucasians deletion of the entire RHD gene is the most common cause of the RhD-negative phenotype, *RHDψ* (66%), *RHD*-*CE*-*D* hybrid gene (15%) and *RHD* gene deletion (18%) are the most prevalent causes of the RhD-negative phenotype among black Africans [1, 6]. Studies have shown that the D-negative phenotype is present in about 10.08% of the Iranian population [7], but there are not any reports identifying the molecular background of D-negative phenotypes in our country. This study for the first time identified the genetic mechanisms of RhD-negative phenotype among the Iranian population. The hybrid *Rhesus box* is a genomic segment with an approximate length of 9000 base, resulting from the *RHD* gene deletion and the integration of two segments of upstream and downstream *Rhesus boxes* that flank the *RHD* gene. This hybrid sequence was used as a marker to identify the *RHD* gene deletion [1]. Regarding the similarity of RhD-negative frequency among Caucasians and Iranian population and high prevalence of *RHD* gene deletion in Caucasians, we decided to evaluate the presence of hybrid *Rhesus box* genomic segment by PCR-SSP and PCR-RFLP. In addition, we performed real-time PCR to investigate the presence of *RHD* gene exons in our samples as a confirmatory test for gene deletion.

## Materials and Methods

### Sample Collection

Blood samples obtained into K3EDTA-containing tubes from 200 RhD-negative randomly selected donors at the Tehran Blood Transfusion Center. This study was approved by the Ethics Committee of the Institute for Educational and Research in Transfusion Medicine.

### Phenotype Determination

The antigenic phenotype (D, C, E, c, e) of red blood cells were determined for all specimens using a monoclonal antibody (IMMUNDIAGNOSTIKA, GmbH, Germany) by standard serological method. Monoclonal antibody reagents were used to test the following specificities: anti-C (RH2, clone MS24), anti-E (RH3, clone MS80/MS258), anti-c (RH4, clone MS33) and anti-e (RH5, clone MS62/MS69).The anti-D reagent was a blend of both IgG and IgM. Subsequently, weak D test was performed on all samples by indirect antiglobulin test (IAT) according to the standard serological guidelines. Adsorption–elution test was not performed in this study.

### DNA Extraction

Genomic DNA was extracted from buffy coat samples using column-based DNA extraction kit (YT 9040, YEKTA TAJHIZ AZMA, IRAN). The extracted DNA was stored at − 20 °C after determining the concentration using the *Nano Drop* (one/one^c^, Thermo Fisher Scientific Inc, USA).

### PCR-SSP

A 2778-bp fragment was amplified from the hybrid *Rhesus box* gene, causing by deletion the *RHD* gene. This segment flanking the breakpoint region in the hybrid *Rhesus box* gene comprised of segments from upstream *Rhesus box* (775 bp), identity region (1467 bp) and downstream *Rhesus box* (536 bp) [1]. The segment was amplified by the forward primer (u1-s) and reverse primer (rnb31) (Table 1). PCR reaction was performed in a volume of 25 μl in 35 cycles using thermal cycler (PEQSTAR 2X 95-08002, PEQLAB Biotechnology GmbH, Germany) under the following conditions: initial denaturation at 95 °C for 10 min, denaturation at 92 °C for 20 s, annealing at 64 °C for 30 s, extension at 68 °C for 3 min, final extension at 72 °C for 5 min. The concentration of forward and reverse primers was 0.4 μm. The PCR products were electrophoresed on 0.8% agarose gel.

**Table 1:** Sequence and specificity of primers comprising segments of hybrid (AJ252313), downstream (AJ252312) and upstream *Rhesus box* (AJ252311) (Reproduced with permission from [1, 8, 9])

Primer	Sequence	Specificity
u1-s	TGAGCCTATAAAATCCAAAGCAAGTTAG	Hybrid and upstream *Rhesus box*
rnb31	CCTTTTTTTGTTTGTTTTTGGCGGTGC	Hybrid and downstream *Rhesus box*
rez7	CCTGTCCCCATGATTCAGTTACC	Hybrid and downstream and upstream *Rhesus box*

### PCR-RFLP

The PCR-RFLP was used to discriminate the status of homo- or heterozygosity of *RHD* gene deletion allele [1]. The primers were designed so that they could amplify both fragments of the hybrid *Rhesus box* in the deletion of *RHD* gene and also of the downstream *Rhesus box* in the presence of *RHD* gene [1, 8, 9]. The sequences of forward (Rez7) and reverse (rnb31) primers are shown in Table 1. PCR reaction was performed as described for PCR-SSP, but the number of cycles was 30 and annealing temperature was 68 °C. The PCR products were digested with *Pst*I enzyme (Jena Bioscience, GmbH, Germany) for 1 h at 37 °C and electrophoresed on 2% agarose gel.

### Real-time PCR

The real-time PCR was used to investigate the presence of three exons 5, 7 and 10 in the *RHD* gene by sequence-specific primers (Table 2). The RHD-positive sample was used as a control for the amplification of exons and melting curve analysis of PCR products. In the presence of *RHD* gene deletion, none of the three exons would be amplified, while in the presence of any *RHD* allele that resulted in RhD-negative phenotype it was expected that one or more of these exons would be amplified based on the type of the allele [10, 11].

**Table 2:** Primers’ sequence of exons 5, 7, and 10 of the *RHD* gene (Reproduced with permission from [12])

Gene	Forward/reverse	Sequence	Product size
Exon 5	F	5′-CGCCCTCTTCTTGTGGATG-3′	82 bp
	R	5′-GAACACGGCATTCTTCCTTTC-3′	
Exon 7	F	5′-CTCCATCATGGGCTACAA-3′	90 bp
	R	5′-CCGGCTCCGACGGTATC-3′	
Exon 10	F	5′-CCTCTCACTGTTGCCTGCATT-3′	74 bp
	R	5′-AGTGCCTGCGCGAACATT-3′	

Real-time PCR reaction was performed in a volume of 25 μl in 30 cycles using thermal cycler (Rotor-Gene, RG3000, Corbett, Australia) and SYBR Green Master Mix under the following conditions: initial denaturation at 94 °C for 2 min, denaturation at 95 °C for 30 s, annealing at 55 °C for 30 s, extension at 72 °C for 30 s. The concentration of forward and reverse primers for each exon was 0.4 μm.

### *RHD*-*CE*-*D* Hybrid Molecular Analysis

We initially analyzed the presence of exons 3, 4, 6 and 9 of *RHD* gene using the PCR-SSP method [13] in one sample whose real-time PCR reaction was positive for exon 10. Then, we investigated the presence of introns 1 and 2 of the *RHD* gene by PCR-RFLP [14, 15].

### Weak D Molecular Analysis

Weak D molecular diagnostic kit (inno-train, Diagnostic GmbH, Germany) was used for further analysis of the positive sample in which the three exons 5, 7 and 10 were amplified by RT-PCR according to the manufacturer’s guidelines. Exon 6 was sequenced (ABI 3130 XL Applied Biosystem, Macrogene, Korea) to confirm the weak D allele type.

## Results

### Rh Antigen Phenotypes

The phenotype of the antigens D, C, E, c, e was determined on samples of 200 blood donors (Table 3). An indirect antiglobulin test was performed for the elimination of donors with weak D phenotype. The result of indirect antiglobulin test proved negative in all donors.

**Table 3:** Frequency of phenotypes and predicted genotypes

Phenotypes	The most probable genotypes	Number(200 samples)	Frequency (%)
D−C−E−c+e+	cde/cde	184	92
D−C+E−c−e+	Cde/Cde	4	2
D−C+E−c+e+	Cde/cde	11	5.5
D−C−E+c+e+	cdE/cde	1	0.5

### Hybrid *Rhesus box* Gene Analysis by PCR-SSP

**Fig. 1:**
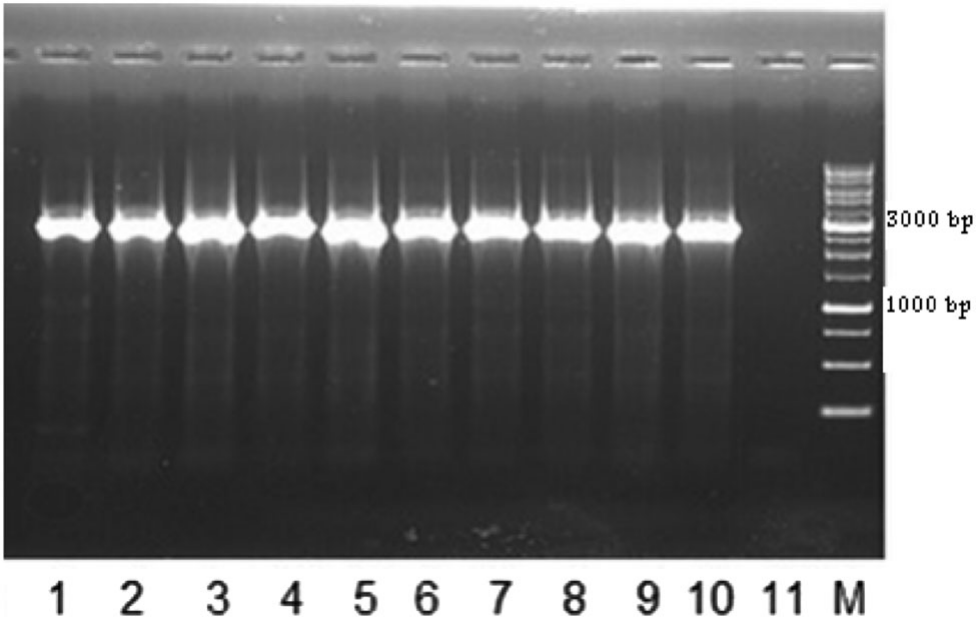
PCR-SSP for hybrid *Rhesus box* gene. A 2778 bp fragment was amplified by PCR-SSP indicates the presence of hybrid *Rhesus box* gene (lanes 1–10). Lane 11: Negative control. M: Molecular marker 1 kb (YT8507, YEKTA TAJHIZ AZMA, IRAN)

The genomic segment with a length of 2778 bp was amplified using PCR-SSP technique, indicating that all 200 (100%) donors were positive for hybrid *Rhesus box* gene in at least one allele (Fig. 1).

### Zygosity Analysis of Hybrid *Rhesus box* Gene by PCR-RFLP

**Fig. 2:**
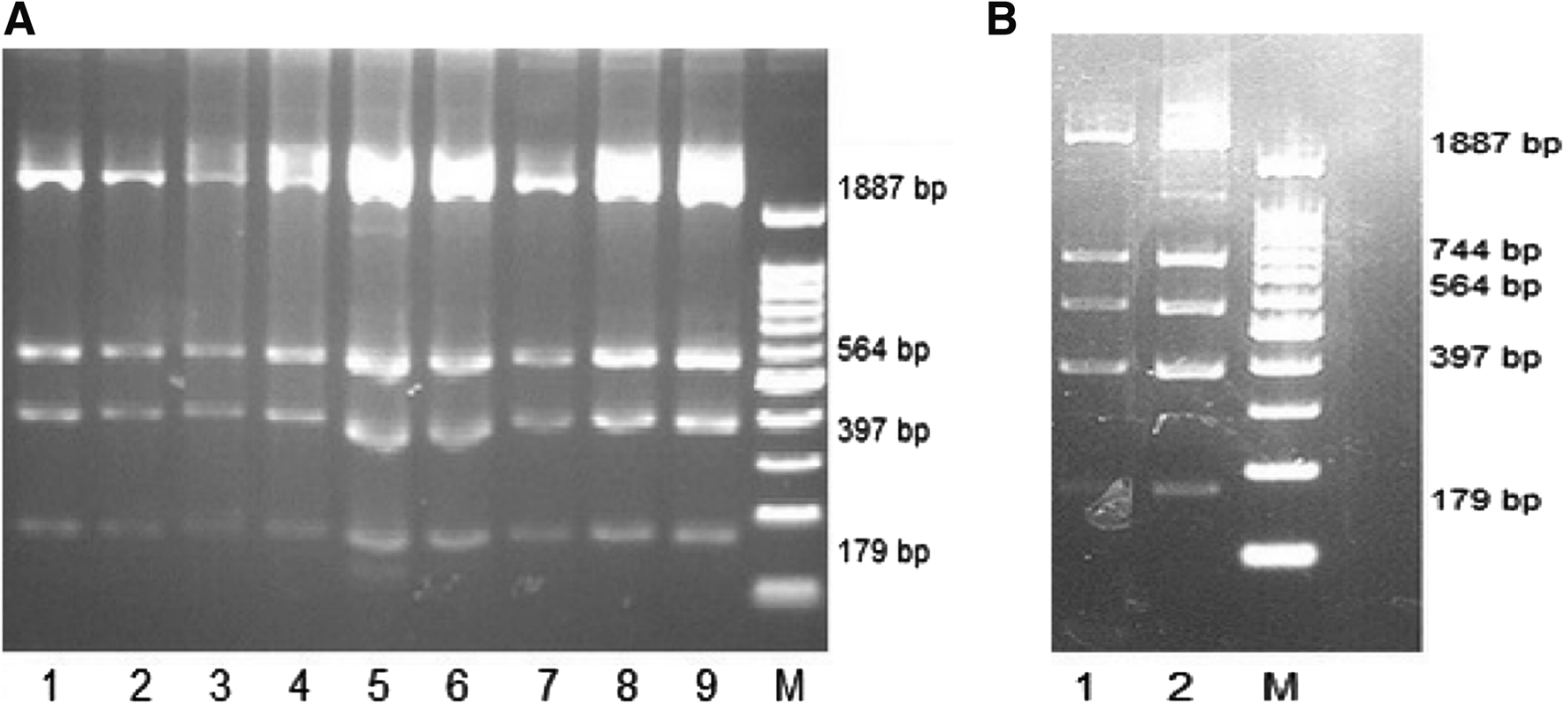
PCR-RFLP analysis for RHD gene deletion. **a** Banding patterns of RHD−/RHD− homozygotes. There are 3 *Pst*I sites in the hybrid *Rhesus box* amplicons. Digestion with this enzyme (*Pst*I) results in 179, 397, 564, 1887-bp fragments (lanes 1–9). **b** PCR-RFLP for RHD+/RHD− heterozygous samples. An additional 744-bp fragment results from downstream *Rhesus box* digestion and indicates the heterozygous situation (lanes 1–2). M: Molecular marker 100 bp (YT8503, YEKTA TAJHIZ AZMA, IRAN)

A genomic segment with an approximate length of 3030 bp was amplified by PCR and specific primers and digested with *Pst*I enzyme. The number and size of fragments on a 2% agarose gel showed that 198 (99%) of donors had a hybrid *Rhesus box* genomic segment in both RH gene alleles (homozygote) (Fig. 2a) and the 2 remaining donors (1%) had a hybrid *Rhesus box* in one of the RH gene alleles (heterozygote); in the latter, in addition to hybrid *Rhesus box,* a genomic segment was amplified from downstream *Rhesus box* of another allele (Fig. 2b).

### *RHD* Exons Analysis

In this study, none of the exons 5, 7 and 10 were amplified in 198 (99%) of 200 samples studied by real-time PCR. Of the two remaining samples (1%), all three exons 5, 7 and 10 were amplified in one sample, and in the other one only exon 10 was amplified. In the positive sample for all three exons 5, 7 and 10 with more molecular analysis by the diagnostic kit, weak D allele type 11 (885 G>T) was detected and confirmed by sequencing of exon 6. In the sample that was positive only for exon 10, the results of PCR-SSP for exons 3, 4, 6 and 9 indicated that these exons were absent in the *RHD* gene in this case. Results of molecular analysis of introns 1 and 2 showed that this sample is a *RHD*-*CE (2*-*9)*-*D*_*2*_ hybrid allele. (Table 4).

**Table 4:** Frequency of *RHD* gene alleles in RhD-negative samples

RHD gene alleles	Number (200 samples)	(%)	Phenotypes	PCR-SSP (for hybrid *Rhesus box*)	PCR-RFLP (for *RHD* gene deletion)	*RHD* exons 5, 7, 10
Gene deletion	198	99	D−C−E−c+e+ (92.92%) D−C−E+c+e+ (0.5%) D−C+E−c−e+ (1.51%) D−C+E−c+e+ (5.05%)	+	Homozygote	–
Weak D type 11	1	0.5	D−C+E−c−e+	+	Heterozygote	+
*RHD*-*CE(2*-*9)*-*D*_*2*_	1	0.5	D−C+E−c+e+	+	Heterozygote	Only 10 +

## Discussion

The prevalence of D-negative phenotype is estimated to be between 15 and 17% among Caucasians, 5% in black Africans and less than 3% in Asians [16]. The prevalence of D-negative phenotype in Iran is estimated at approximately 10.08% [7], which is more consistent with the Caucasian population. This study for the first time identified the genetic mechanisms of D-negative phenotype among the Iranian population.

Our results, using the PCR-SSP, showed that all donors were positive for the presence of a 2778 bp genomic segment obtained from the hybrid *Rhesus box* gene. The amplification of this segment implies that all donors have at least one *RHD* gene deletion allele, and another allele of the *RHD* gene may be a deletion or non-deletion allele. Therefore, the PCR-RFLP was used to check the status of the other allele for the presence or absence of *RHD* gene.

The results of PCR-RFLP showed that 198 (99%) of donors in both RH gene alleles had a hybrid *Rhesus box* genomic segment, which means that these cases are homozygote for *RHD* gene deletion. Analysis of exons 5, 7 and 10 using real-time PCR also showed that none of the three exons were amplified in these 198 donors, and the PCR-SSP and PCR-RFLP results were confirmed. Moreover, the PCR-RFLP results showed that 2 (1%) of the RhD-negative donors had a *RHD* gene allele. The analysis of exons 5, 7 and 10 using real-time PCR showed that all three exons 5, 7 and 10 were amplified in one sample, but, in the other one, only exon 10 was amplified. In the first sample, further molecular analysis showed that the donor had a weak D allele type 11 that has not been detected by the weak D serological test. In some cases, the weak D phenotype may not be detected by the conventional serological tests (indirect anti-globulin test) [17]. The 885 G>T mutation associated with the replacement of M295I results in lowered density of D antigen on red blood cells in the weak D type 11 [18]. In the second sample that was positive only for exon 10, the results of further molecular analyses for exons 3, 4, 6 and 9 and introns 1 and 2 of *RHD* gene indicated the presence of the *RHD*-*CE (2*-*9)*-*D*_*2*_ hybrid allele. Several studies in East Asia have shown that the most common mechanisms for the D-negative phenotype in this population are the *RHD* gene deletion, the DEL allele (approximately 10–30%) and the *RHD*-*CE*-*D* hybrid allele (approximately 10%) [19, 20]. These genetic backgrounds of RhD-negative are different from the Iranian population located in the Middle East, while the frequency of our results is more similar to the European population [21]. The frequency of D-negative phenotype with a non-deletion *RHD* gene allele is approximately 0.6% in Caucasians, 10% in black Africans and 30% in Asians [2, 22]. In our study, non-deletion *RHD* gene allele was found in 2 (1%) D-negative donors, indicating more consistency with the Caucasian population. Genotyping of RhD-negative donors at the blood transfusion centers is very important because some alleles like DEL and weak D may not be detected by conventional serological tests and are considered as RhD-negative. Transfusion of these blood products into RhD-negative individuals, especially for girls and women with childbearing potential, may increase the risk of immunization with anti-D in these individuals. So, the screening of RhD-negative donors by genotyping for identification of *RHD* gene alleles, especially in a population where these alleles are highly prevalent, can be very important [17, 23]. Despite the high prevalence of DEL allele in East Asia (approximately 10–30% of the RhD-negative population), our results (high frequency of *RHD* gene deletion) imply that the frequency of the DEL allele among the Iranian population should be very low and similar to the Caucasian population [21, 24]. In our study, the *RHD* gene deletion had the highest association with the D−C−E−c+e+ phenotype (92.92%) and 87.5% of the C+/E+ donors had *RHD* gene deletion alleles. The frequency of D-negative phenotype with the presence of *RHD* gene non-deletion alleles in association with C and E antigens has also been reported [2, 11]. Both donors with non-deletion allele in this study were positive for C antigen.

## Conclusion

In this study, using PCR-SSP, PCR-RFLP, and real-time PCR to identify the genetic background of D-negative phenotype showed that *RHD* gene deletion is the most common genetic mechanism for D-negative phenotype in Iran. The results of the present study are consistent with other studies performed in Caucasians. Furthermore, hybrid *Rhesus box* molecular analysis could effectively identify this allele.
